# Supplementary material for: Predicting the structure of large protein complexes using AlphaFold and Monte Carlo tree search
Source: Nat Commun. 2022 Oct 12;13:6028. doi: 10.1038/s41467-022-33729-4 (PMC9556563; doi:10.1038/s41467-022-33729-4)
Supplement: Supplementary file 1 — Supplementary Information [file 41467_2022_33729_MOESM1_ESM.pdf]

Supplementary Information

Supplementary figures

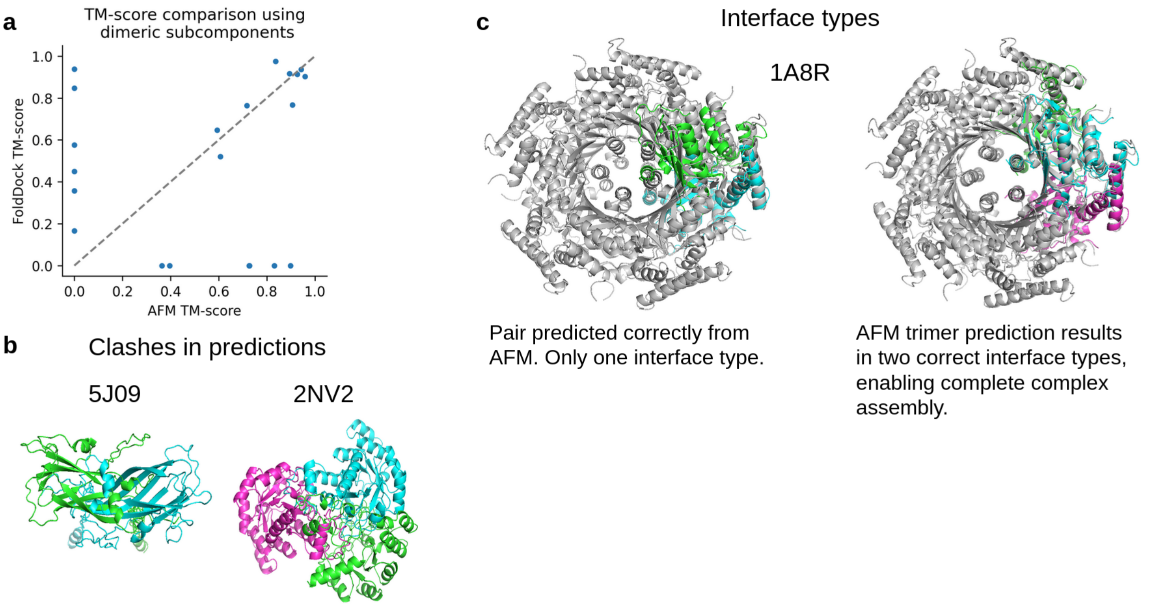

Supplementary Figure Figure 1. a) TM-scores for the **21** complexes that could be assembled to completion using pairwise interactions with AFM or FoldDock (AF) respectively. **b)** Clashes in the predictions are shown for pairwise and trimeric interactions belonging to the complexes 5J09 and 2NV2 respectively. **c)** Assembly overlaps occur due to only one possible configuration being predicted. PDB ID 1A8R, is a homo 10-mer, containing only one unique chain (A10). This means that all interactions are between copies of this chain. When predicting pairwise interactions, only one conformation is found. This can be circumvented by predicting trimeric interactions.

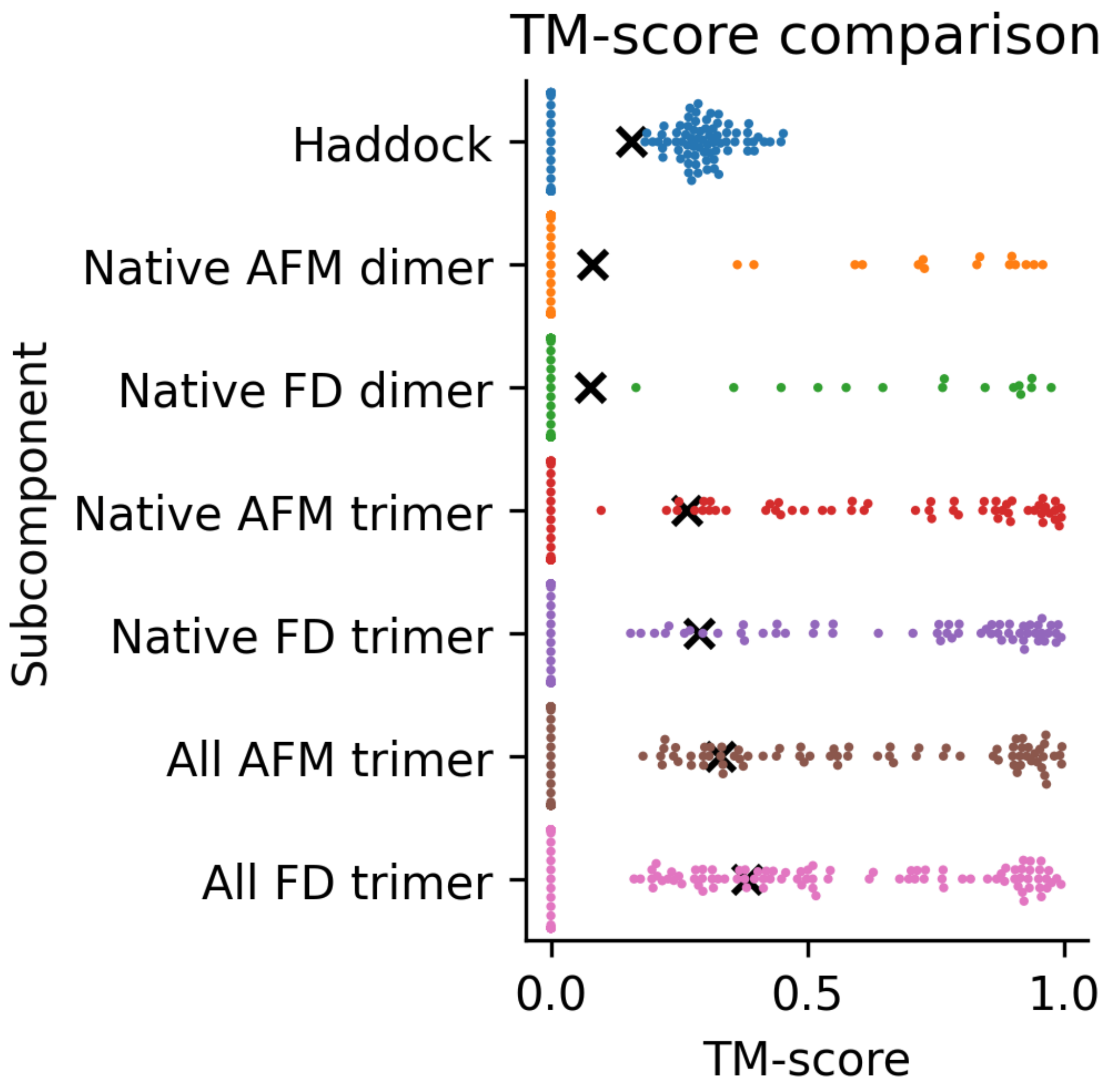

Supplementary Figure 2. TM-scores for the complexes that could be assembled to completion using Haddock with native chain structures, FoldDock (FD) or AFM and predicted native dimeric, native trimeric and all trimeric subcomponents, respectively. The complete set of complexes from the three approaches (n=140) is shown, with scores of zero representing missing complexes for each approach. The points display the TM-score of the individual complexes and the black “x” marks the average scores. The average TM-scores are 0.16, 0.08, 0.08, 0.27, 0.29, 0.33 and 0.38 from top to bottom, respectively.

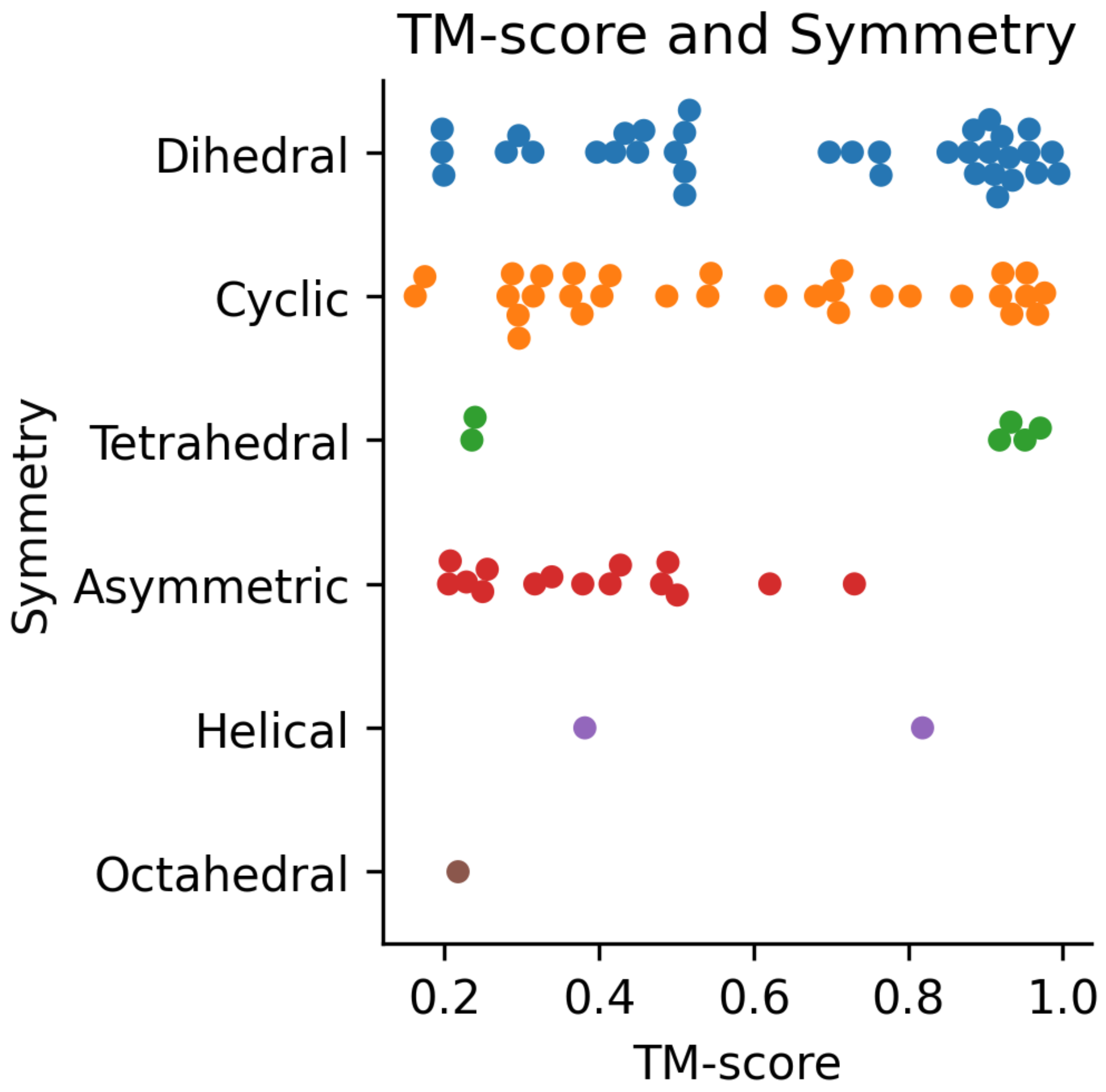

Supplementary Figure 3. TM-score and symmetry for the complete assembled complexes (n=91) using all possible trimers predicted with FoldDock. The number of assembled complexes per symmetry are: Dihedral: 36, Cyclic: 31, Tetrahedral: 6, Asymmetric: 15, Helical: 2 and Octahedral: 1. The median TM-scores are: Dihedral: 0.75, Cyclic: 0.54, Tetrahedral: 0.93, Asymmetric: 0.38, Helical: 0.60, Octahedral: 0.22.

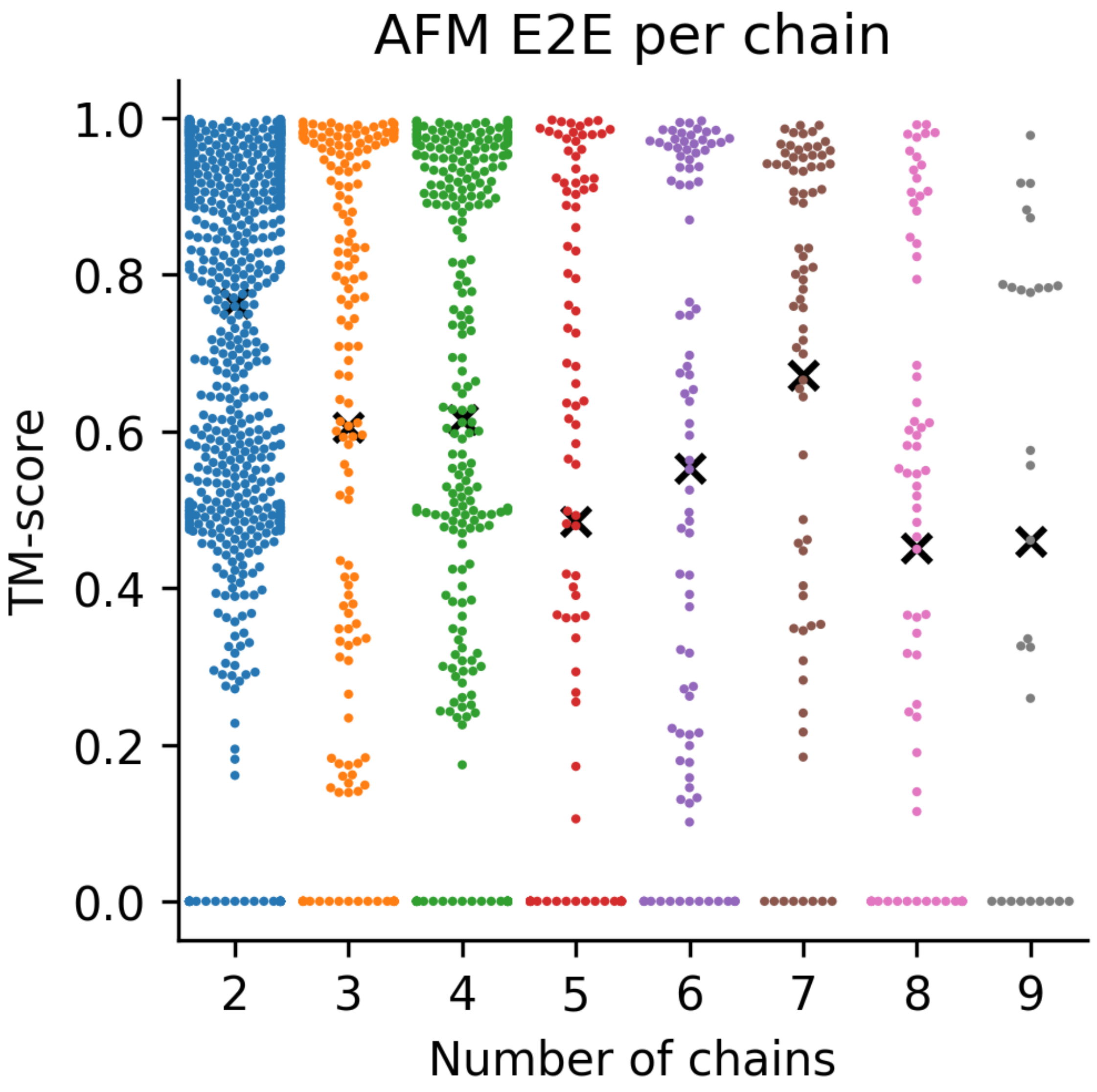

Supplementary Figure 4. TM-score for AFM E2E on a dataset without homology to the AFM training set consisting of 931 dimers, 164 trimers, 269 tetramers, 103 pentamers, 91 hexamers, 74 heptamers, 73 octamers and 28 nonamers. The black “x” marks the average for each oligomer and each point represents one sample. The samples at 0 are those that failed due to memory limitations. The average score decreases with the number of chains.

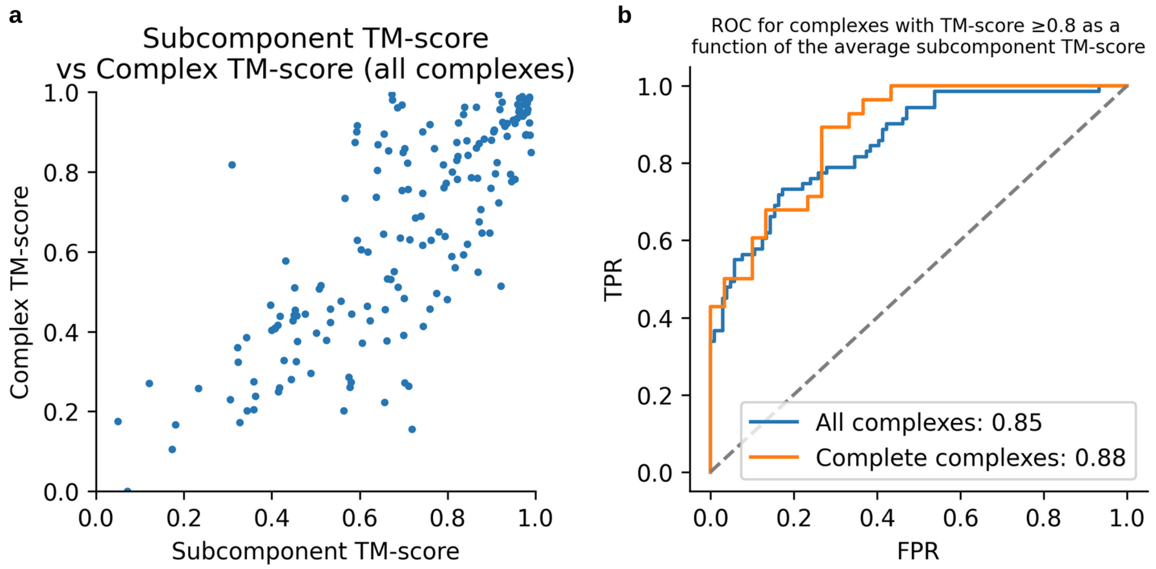

Supplementary Figure 5. a) Average TM-score of subcomponents vs TM-score of the whole complex for all assemblies (n=58) using the native trimers predicted with FoldDock. When the subcomponents display high accuracy, so does the assembled complexes (Spearman R = 0.79). **b)** ROC curve, where positives are assemblies of TM-score ≥0.8, as a function of the avergae subcomponent TM-score using all and only the complete assemblies, respectively. The AUC is 0.85 for all complexes (positives = 71, negatives =104) and 0.88 for the complete assemblies (positives = 28, negatives = 30).

Supplementary tables

Supplementary Table 1. MedianTM-score and the number of complexes assembled to completion for AlphaFold-multimer (AFM) and FoldDock using different subcomponents.

Method	Subcomponent	Median TM-score	Complete assemblies out of 175 in total
AFM	Native dimer	0.83	15
FoldDock	Native dimer	0.77	15
AFM	Native trimer	0.74	55
FoldDock	Native trimer	0.88	58
AFM	All trimer	0.61	74
FoldDock	All trimer	0.51	91

Supplementary Table 2. Average TM-score and the number of complexes assembled to completion across all approaches for AlphaFold-multimer (AFM) and FoldDock.

Method	Subcomponent	Average TM-score	Complete

			assemblies out of 175 in total
AFM	Native dimer	0.10	113
FoldDock	Native dimer	0.09	113
AFM	Native trimer	0.33	113
FoldDock	Native trimer	0,36	113
AFM	All trimer	0.41	113
FoldDock	All trimer	0.47	113
